# Vaginal Sparing Radiotherapy Using IMPT and Daily Dilator Placement for Women with Anal Cancer

**DOI:** 10.14338/IJPT-21-00025

**Published:** 2022-04-26

**Authors:** Scott C. Lester, Laura A. McGrath, Rachael M. Guenzel, Jenae C. Quinn, Carolyn J. Schultz, T. Baron Bradley, Bret D. Kazemba, Shima Ito, Christopher L. Hallemeier

**Affiliations:** Department of Radiation Oncology Mayo Clinic, Rochester, MN

**Keywords:** anal cancer, proton therapy, sexual dysfunction, women's health

## Abstract

Sexual dysfunction is a common toxicity and detrimental for the quality of life of women treated with chemoradiotherapy for anal cancer. Sexual dysfunction occurs because the vagina is closely approximated to the anal canal and typically receives substantial doses of radiation. Strategies for mitigation have largely been focused on posttreatment therapy and symptom management. The use of daily vaginal dilator placement during radiotherapy to mitigate dose to the vagina has been previously explored with modest gains, while proton therapy is under active investigation for the treatment of anal cancer. Use of proton therapy for anal cancer reduces dose to some organs at risk but may inadvertently increase vaginal toxicity if the proton beam terminates in the vaginal tissue. Herein, we present the case histories of 2 women treated for squamous cell carcinoma of the anal canal with the novel combination of intensity-modulated proton therapy and daily vaginal dilator placement to maximally reduce dose to the vagina and protect it from areas of increased energy deposition at the end of the proton range.

## Clinical Scenarios

This case series has been deemed exempt by the Mayo Clinic Institutional Review Board. Patient A was a 78-year-old female diagnosed with clinical stage T2, N0 moderately differentiated squamous cell carcinoma of the anal canal. A pelvic magnetic resonance imaging scan and exam concordantly identified a 2.3-cm mass. The mass involved the right aspect of the upper 1.2 cm of the internal anal sphincter and directly abutted the adjacent rectal wall. She was treated with 5400 cGy to the primary site and 4500 cGy to the mesorectum and lymph nodes, both in 30 fractions with concomitant mitomycin-c and 5-fluorouracil.

Patient B was a 72-year-old female diagnosed with clinical T2, N0 squamous cell carcinoma of the anal canal. She was treated with 5000 cGy to the primary site and 4250 cGy to the mesorectum and lymph nodes, both in 25 fractions with concomitant mitomycin-c and 5-fluorouracil.

## Simulation

**Figure 1.**
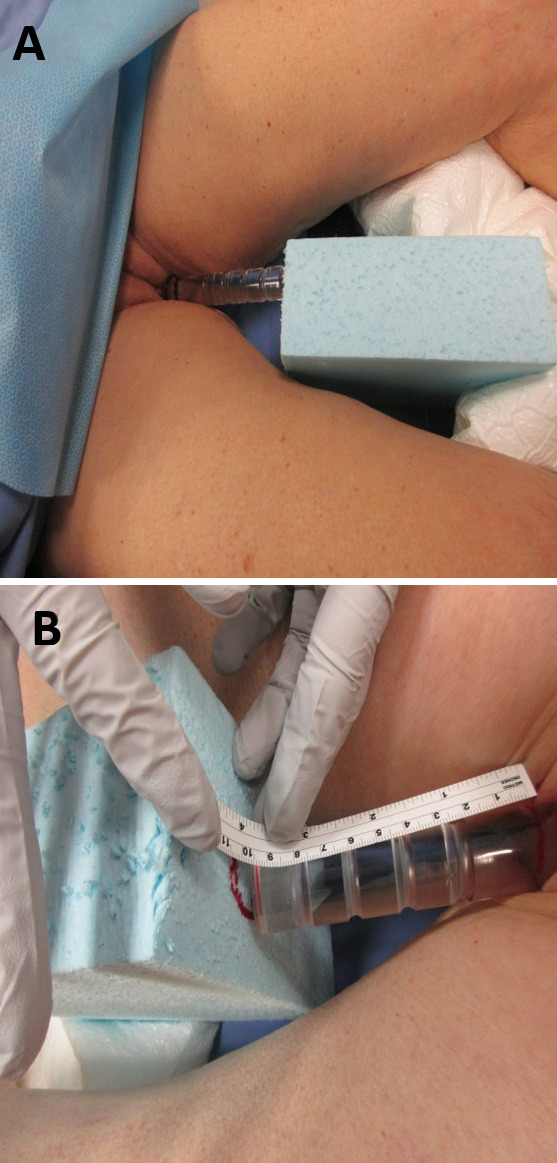
(A) Patient A, demonstrating centrally indexed foam block and (B) Patient B, focused view demonstrating initial marking and measurements to achieve reproducibility.

Both patients were treated with a novel technique of intensity modulated proton therapy with daily placement of a vaginal dilator during radiotherapy. Each patient presented to simulation with a full bladder as instructed. They were positioned supine with the thighs abducted into the frog leg position. A custom vacuum immobilization device (Vac-Lok, Civco, Iowa) was used to immobilize the lower extremities. A foam block was indexed centrally in the immobilization device (**Figure 1A**). An acrylic vaginal dilator (Dynamic Group Precision Molds and Molding, Ramsey, Minnesota) was lubricated and inserted into the vagina. A size 7/8-inch diameter dilator was used with the intent to comfortably displace the anterior vaginal tissue. We specifically aimed to avoid maximally stretching the circumference of the vagina to limit exacerbating potential radiation-induced mucosal injury during radiotherapy. The point of contact with the external genitalia was marked with a permanent marker on the dilator and measured to ensure a reproducible depth of insertion (**Figure 1B**). The patient was slid gently down until the end of the dilator firmly approximated the foam block. A target identifying the region of contact was marked on the foam to ensure a reproducible dilator angle. A single radio-opaque marker was placed at the anal verge. Careful attention was paid to ensure the posterior soft tissues were flat and free of skin folds. A computed tomography scan was acquired to image the pelvis with the dilator in place. A second identical scan with intravenous contrast was acquired. Last, the dilator was removed, and a third scan was acquired.

## Contouring and Planning

**Figure 2.**
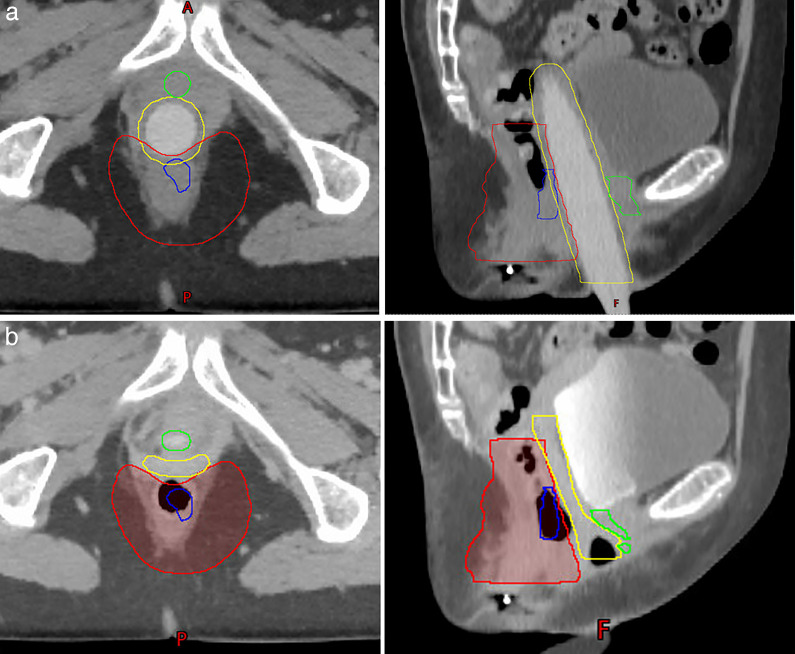
Patient B, (A) with dilator and (B) without dilator blue = GTV, red = CTV5000, yellow = vagina, green = urethra (contours rigidly transformed to scan b).

Contouring was done per standard of care with the gross target volume delineated and expanded by 25 mm to a high-dose clinical target volume (CTV). The CTV expansion did not include the dilator that immediately abuts the anus. The typical at-risk areas were covered with the elective region. Patient A was treated with a small additional expansion into the dilator to account for potential placement uncertainty, while Patient B did not have this expansion included. A uniform expansion of 3 mm out from the dilator was used to estimate dose to the vaginal mucosa. No attempt was made to exclude the CTV or dose from the posterior aspect of the vaginal mucosa structure given the proximity to tumor and imprecise construction of this organ at risk. The gross target volume, CTV, vagina with the dilator in place, and urethra are shown in **Figure 2A** for patient B. Target volumes were rigidly deformed on to a second scan without the dilator in place (**Figure 2B**). This was done to facilitate plan evaluation had the patient presented unable to place the dilator during radiotherapy. It is important to note that the dilator enables relatively accurate delineation of the rectovaginal septum and in practice it is difficult to exclude the vagina from the CTV without a dilator in place. Furthermore, the rigid CTV does not fully encompass the anterior anus without the dilator in place so the benefits of dosimetric benefits sparing may be greater than what is shown with a rigid comparison.

## Planning

**Figure 3.**
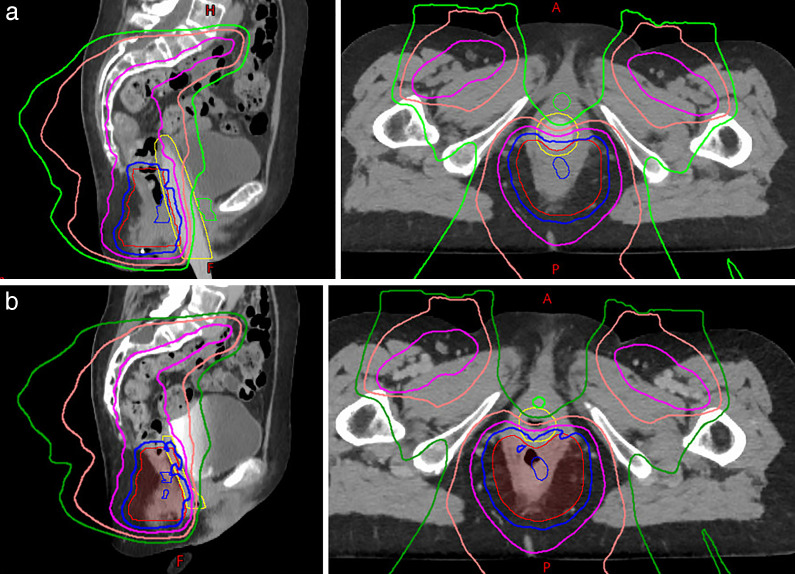
Patient B isodose lines as follows: 5000 cGyE (blue), 4250 (magenta), 2500 (light pink), and 1250 (green). (A) With the dilator inserted, the lower anterior vagina including the anterior introitus and urethra receives < 1250 cGy. (B) Without the dilator in place, the vagina collapses back toward the target and the majority is exposed to > 75% of the prescription. In addition, dose to the posterior bladder and urethra are increased.

**Figure 4.**
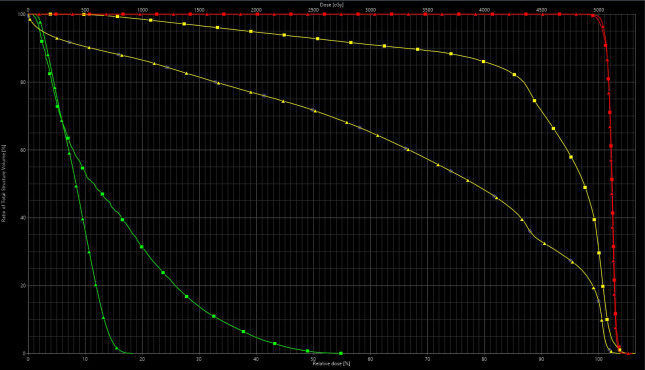
DVH demonstrating selected structures (ctv in red) and a comparison of dose to vagina (yellow) and urethra (green) with (triangle) and without (square) the dilator in place. Relevant doses achieved for the plan with the dilator in place and delivered to the patient were as follows: bladder mean 22.4 Gy, bone marrow V10 Gy < 65.8%, small bowel V30Gy 84cc, V15 Gy 140 cc, femoral heads V30 approximately 2% for each, and external genitalia mean 1.6 Gy. Abbreviation: DVH, dose-volume histogram.

**Figure 5.**
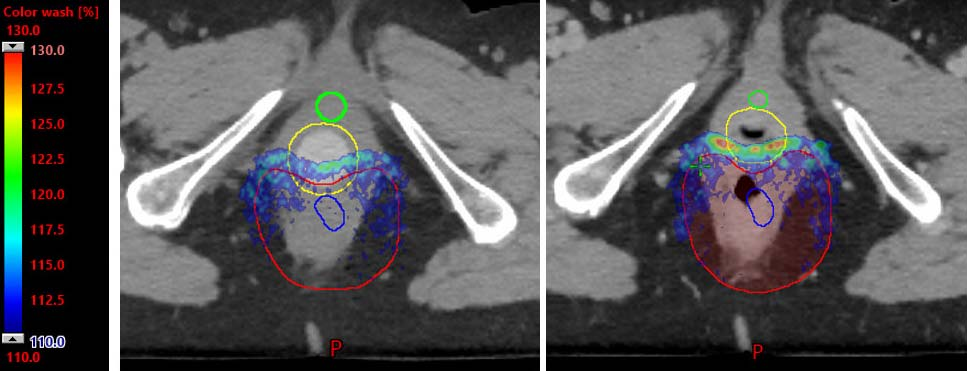
LET-weighted model set to 110% to 130% of prescription estimating the high LET end of range (A) with and (B) without dilator. In the absence of the dilator, this high LET area is deposited within the vaginal mucosa and could increase toxicity. Abbreviation: LET, linear energy transfer.

Both patients' treatments were planned with a 3-field technique using an anterior beam and 2 posterior oblique beams with the Eclipse treatment planning system (version 13.7.15; Varian, Palo Alto, CA, USA) using multifield optimization. The beam specific target volume of the anterior field only covered superficial inguinal lymph nodes to protect the anterior vagina, and 2 posterior oblique beams covered the entire targets. Target coverage was robustly optimized to 5 mm of setup uncertainty and 3% of range uncertainty. The dilator was estimated at 285 HU. **Figure 3** shows representations of the treated plan and **Figure 4** shows a corresponding dose volume histogram for select organs at risk. In addition, an in-house linear energy transfer (LET)–weighted Monte Carlo model was used to estimate areas of elevated LET. These increases occur in the region of and immediately beyond the Bragg peak and may correspond with areas of heightened toxicity from proton therapy. A stated plan goal was to maximize the LET that is deposited into the dilator (so called *LET-sink*) and avoid the vaginal mucosa (**Figure 5**).

## Verifications

Both patients underwent weekly verification computed tomography scans in the treatment position to ensure the plan goals were safely maintained.

### Outcome

Neither patient at any point complained of mucosal pain, discomfort, or vaginal bleeding during treatment. Overall treatment tolerance was good with nonhematologic side effects limited to Grade 1 to 2 without treatment interruption or hospitalization. Both patients are now more than 12 months out from treatment and have complete clinical response. Patient A reports healthy bowel function. Patient B reports 1 to 2 bowel movements a day with occasional tinges of blood on the stool when constipated. Neither patient reports fecal incontinence, urinary symptoms, or other radiation-related changes. Neither patient was sexually active before treatment nor has engaged in intercourse since treatment.

## Discussion

Over the past decade, extensive efforts have been made to develop a displacement technique for men undergoing radiotherapy for prostate cancer [1]. Gel-based displacement of the prostate comes at significant cost and requires an invasive procedure with a risk for rectal injury [2, 3]. In contrast, vaginal displacement is achieved without a procedure or risk to adjacent organs and cost is minimal. Yet, vaginal displacement for women undergoing radiotherapy for anal cancer is infrequently reported and we believe infrequently used among practicing radiation oncologists [4, 5]. This is likely a consequence of under reporting of sexual toxicity. Several reports of modern radiotherapy for anal cancer including one from our institution omit sexual dysfunction entirely from toxicity analyses [6–8]. It is well-understood that collateral radiation dose to the vagina can cause dryness, mucosal thinning, vaginal shortening, and vaginal fibrosis [9, 10]. The caudal vagina, which includes the introitus, is thought to be particularly sensitive compared with the rostral vagina. These functional and anatomic changes precipitate sexual dysfunction that can diminish quality of life for anal cancer survivors. Herein, we present our most updated technique for maximal sparing of the anterior and distal vagina from radiation effects for 2 women treated for anal cancer. We believe this technique holds promise for women undergoing pelvic radiotherapy for anal cancers as well as lower rectal cancers.

**Figure 6.**
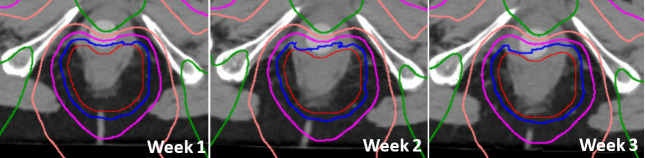
Patient B, weekly verifications demonstrating adequate target coverage and persistent anterior vaginal sparing. Isodose lines as follows: 5000 cGy (blue), 4250 (magenta), 2500 (light pink), and 1250 (green).

Reproducibility and patient tolerance are paramount and interdependent. Weekly verification scans were performed for both patients and plan goals were retained. No adaptive replanning was needed for either patient. Aside from dosimetric advantages, we believe the dilator increased the reproducibility of the internal anatomy by stabilizing the rectum, vagina, and bladder interfaces. As such we have also used this technique to stabilize the vaginal cuff for postoperative radiotherapy of endometrial cancer patients using proton therapy. For Patient A, dilator placement was performed daily initially by the treating doctor and subsequently the treating nurse practitioners. This enabled frequent patient interaction to monitor treatment and dilator tolerance. For Patient B, the same team initially placed the dilators but after 1 week this duty was transferred to a designated radiation therapist. Subsequent verifications demonstrated excellent reproducibility as shown in **Figure 6**.

This technique maximizes the physical advantages of proton therapy, while also neutralizing the potentially toxic high LET from protons that would otherwise put the end of range into the vaginal mucosa. This report is limited by its small sample size and absence of comparative sexual outcomes with alternative treatment techniques. In addition, the LET-weighted model represents an estimation based on potential relative biological effectiveness but may not be generalizable to anal cancer. Last, both patients had small volume primary tumors without vaginal invasion and no prior history of gynecologic surgeries. As such these patients were well-selected for daily dilator placement, but for women with large tumors and/or surgically altered anatomy this approach may not be feasible. It is important to recognize that even in the absence of proton therapy; a vaginal dilator can still reduce the dose to the anterior vagina and should be considered. We applaud the recent opening of the DILANA trial (NCT04094454), which is a randomized trial comparing the effect of tampon diameter on 12-month vaginal fibrosis and should further define the role of vaginal displacement in anal cancer treatment [11].

## References

[1] Miller LE, Efstathiou JA, Bhattacharyya SK, Payne HA, Woodward E, Pinkawa M (2020). Association of the placement of a perirectal hydrogel spacer with the clinical outcomes of men receiving radiotherapy for prostate cancer: a systematic review and meta-analysis. *JAMA Netw Open*.

[2] Levy JF, Khairnar R, Louie AV, Showalter TN, Mullins CD, Mishra MV (2019). Evaluating the cost-effectiveness of hydrogel rectal spacer in prostate cancer radiation therapy. *Pract Radiat Oncol*.

[3] Aminsharifi A, Kotamarti S, Silver D, Schulman A (2019). Major complications and adverse events related to the injection of the SpaceOAR hydrogel system before radiotherapy for prostate cancer: review of the manufacturer and user facility device experience database. *J Endourol*.

[4] Briere TM, Crane CH, Beddar S, Bhosale P, Mok H, Delclos ME, Krishnan S, Das P (2012). Reproducibility and genital sparing with a vaginal dilator used for female anal cancer patients. *Radiother Oncol*.

[5] Mitchell MP, Abboud M, Eng C, Beddar AS, Krishnan S, Delclos ME, Crane CH, Das P (2014). Intensity-modulated radiation therapy with concurrent chemotherapy for anal cancer: outcomes and toxicity. *Am J Clin Oncol*.

[6] Mitra D, Hong TS, Horick N, Rose B, Drapek LN, Blaszkowsky LS, Allen JN, Kwak EL, Murphy JE, Clark JW, Ryan DP, Cusack JC, Bordeianou LG, Berger DL, Wo JY (2017). Long-term outcomes and toxicities of a large cohort of anal cancer patients treated with dose-painted IMRT per RTOG 0529. *Adv Radiat Oncol*.

[7] Jethwa KR, Day CN, Sandhyavenu H, Gonuguntla K, Harmsen WS, Breen WG, Routman DM, Garda AE, Hubbard JM, Halfdanarson TR, Neben-Wittich MA, Merrell KW, Hallemeier CL, Haddock MG (2021). Intensity modulated radiotherapy for anal canal squamous cell carcinoma: a 16-year single institution experience. *Clin Transl Radiat Oncol*.

[8] Ghareeb A, Paramasevon K, Mokool P, van der Voet H, Jha M (2019). Toxicity and survival of anal cancer patients treated with intensity-modulated radiation therapy. *Ann R Coll Surg Engl*.

[9] Son CH, Law E, Oh JH, Apte AP, Yang TJ, Riedel E, Wu AJ, Deasy JO, Goodman KA (2015). Dosimetric predictors of radiation-induced vaginal stenosis after pelvic radiation therapy for rectal and anal cancer. *Int J Radiat Oncol Biol Phys*.

[10] Mirabeau-Beale K, Hong TS, Niemierko A, Ancukiewicz M, Blaszkowsky LS, Crowley EM, Cusack JC, Drapek LC, Kovalchuk N, Markowski M, Mapolitano B, Nyamwanda J, Ryan DP, Woflgang J, Kachnic LA, Wo JY (2015). Clinical and treatment factors associated with vaginal stenosis after definitive chemoradiation for anal canal cancer. *Pract Radiat Oncol*.

[11] Arians N, Häfner M, Krisam J, Lang K, Wark A, Koerber SA, Hommertgen A, Debus J (2020). Intrafractional vaginal dilation in anal cancer patients undergoing pelvic radiotherapy (DILANA) - a prospective, randomized, 2-armed phase-II-trial. *BMC Cancer*.

